# The complete chloroplast genome of *Rhamnus globosa* (Rhamnaceae)

**DOI:** 10.1080/23802359.2020.1791010

**Published:** 2020-07-15

**Authors:** Yanping Xie, Ze Wang, Xianfeng Jiang, Xingwang Zhang

**Affiliations:** a School of Life Sciences, Huaibei Normal University, Huaibei, China; b College of Life Sciences, Henan Normal University, Xinxiang, China; c College of Agriculture and Bioscience, Dali University, Dali, China; d School of Information, Huaibei Normal University, Huaibei, China

**Keywords:** Chloroplast genome, *Rhamnus globosa*, phylogenetic analysis

## Abstract

*Rhamnus globosa* Bunge is a small shrub that can be used in the treatment of swollen poison, because of the antimicrobial activity. Here, we constructed the complete chloroplast genome of the *R. globosa* using Illumina sequencing technology. The circular cp genome was 160,642 bp in size, and comprises a large single-copy (LSC) region of 88,889 bp, a small single-copy (SSC) region of 18,897 bp, and a pair of inverted repeats (IR) of 26,428 bp. The GC content was 37.14% overall, with 35.01%, 31.42%, and 42.75% for the LSC, SSC, and IR regions, respectively. The plastome comprised 129 unique genes including 81 protein-coding genes, 30 tRNAs, and 4 rRNAs. The ML phylogenetic analysis based on 44 chloroplast genomes in Rosales showed a strong sister relationship with *Berchemia* species.

The *Rhamnus* species are famous as dyestuff plants to dye ‘Chinese green’ and medical plants for long time in China. *R. globosa* Bunge is small shrub, formating understories of forests and thickets, slopes below an altitude of 1600 m. It distributes in Anhui, Gansu, Hebei, southern and western of Henan, Hunan, Jiangsu, Jiangxi, Liaoning, southwestern Shaanxi, Shandong and Shanxi in China. They are commonly used for making lubricating oil from the seeds, and for making a green dye from the bark, fruit, and roots. Furthermore, *R. globosa* is sometimes used in Chinese medicine for the treatment of swollen poison (Al-Judaibi and Al-Yousef 2014). However, the plastid genome sequence of this species remains unknown, therefore we report the complete chloroplast (cp) genome of *R. globosa* and present its value of phylogenetic analysis.

Fresh leaves of *R. globosa* for total genomic DNA extraction were collected from Huangcangyu, Anhui Province, China (32.34°N, 117.99°E). The voucher specimen (voucher accession number HCY2019026) was stored at the Key Laboratory of Plant Resource and Biology in Huaibei Normal University. The qualified PCR-amplified library was sequenced with the Illumina NovaSeq Tenplatform (Nanjing Genepioneer Biotechnologies Inc., Nanjing, China). The cp genome was assembled using the program NOVOPlasty 2.7.2 (Dierckxsens et al. 2016). Annotation was performed using GeSeq (Tillich et al. 2017), followed by manual correction for start and stop codons of protein-coding genes. The assembled complete chloroplast genome sequence of *R. globosa* was submitted to NCBI, and the accession number is MT360052.

The complete chloroplast genome of *R. globosa* was 160,642 bp, consisting of a large single-copy (LSC) region of 88,889 bp, a small single-copy (SSC) region of 18,897 bp, and a pair of inverted repeats (IR) of 26,428 bp. The GC content was 37.14% overall, with unevenly distribution across regions of the cp genome, which were found to be 35.01%, 31.42%, and 42.75% for the LSC, SSC, and IR regions, respectively. The plastome comprised 129 unique genes including 81 protein-coding genes, 30 tRNAs, and 4 rRNAs. Nine genes (*ndhA*, *ndhB*, *petB*, *petD*, *atpF*, *rpl16*, *rpl2*, *rps16*, *rpoC1*) contained only one intron and three genes (*rps12*, *clpP* and *ycf3*) contained two introns. Severn protein-coding genes, seven tRNAs, and all four rRNAs were completely duplicated within IRs.

To further investigate its phylogenetic position, a maximum likelihood tree was constructed based on complete chloroplast genome sequences of 44 species in Rosales by online RAxML BlackBox software (Stamatakis et al. 2008), after the sequences were aligned using MAFFT v7.307 (Katoh and Standley 2013). Our results suggested *R. globosa* was close to the other *Rhamnus* species, and sister to *Berchemia* (Figure 1). The current phylogenetic relationship was consistent with the latest results based on cp genome sequences (Zhu et al. 2019; Li et al. 2020). This published *R. globosa* chloroplast genome might provide useful information for phylogenetic and evolutionary studies in Rhamnaceae and Rosales.

**Figure 1.**
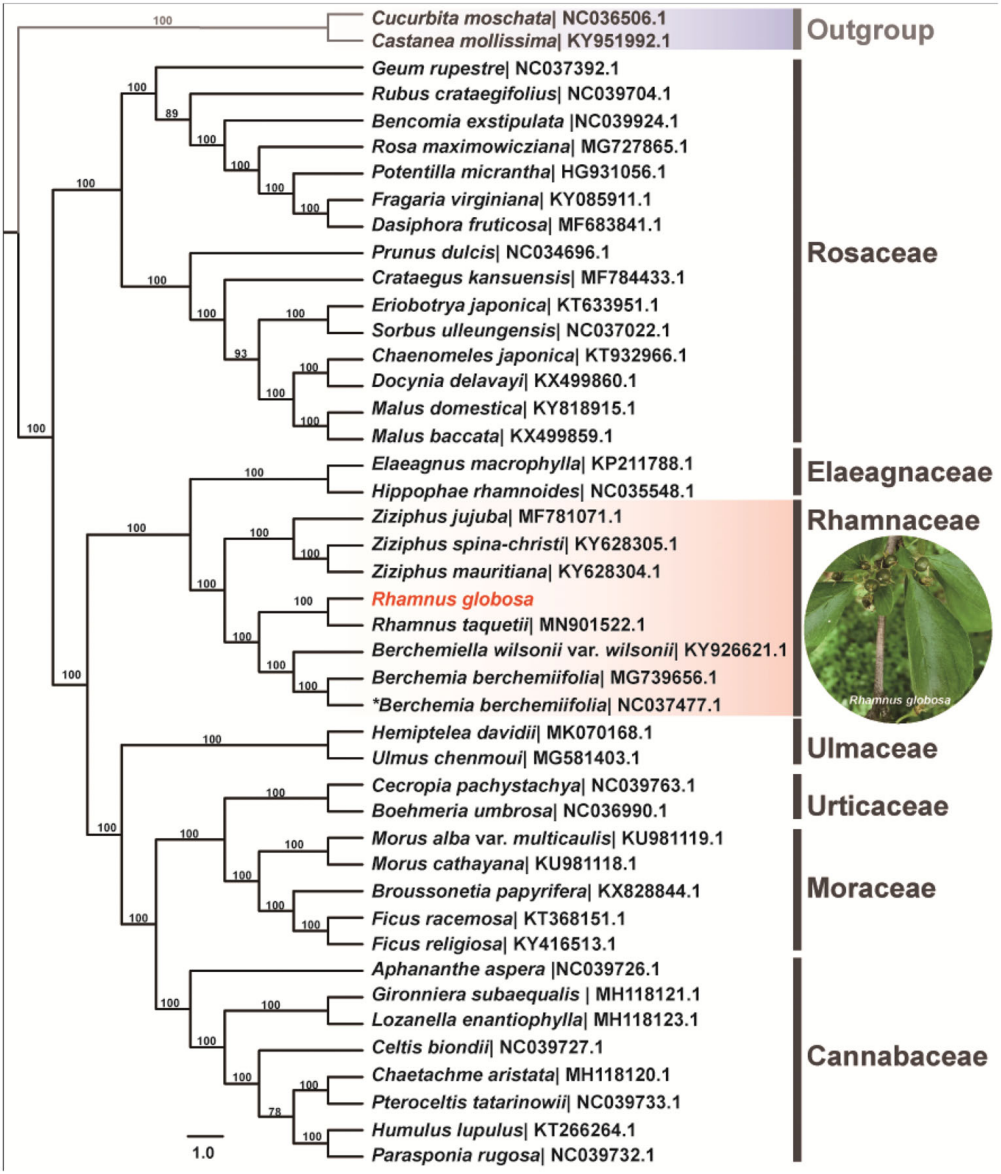
Maximum likelihood tree showing the relationship among *Rhamnus globora* and representative species within Rosales. Bootstrap values are indicated for each branch based on 1000 replicates. The species name marked with asterisk symbol is *Berchemia berchemiifolia* in NCBI, which is the synonym of *Berchemiella berchemiifolia*.

## Data Availability

The complete chloroplast genome sequence and annotation of *R. globosa* that support the findings of this study are openly available in Zenodo at https://zenodo.org/record/3903976#.XvFyVhN9Hn0.

## References

[CIT0001] Al-Judaibi A, Al-Yousef F. 2014. Antifungal effect of ethanol plant extracts on *Candida* sp. Am J Agric Biol Sci. 9(3):277–283.

[CIT0002] Dierckxsens N, Mardulyn P, Smits G. 2016. NOVOPlasty: de novo assembly of organelle genomes from whole genome data. Nucleic Acids Res. 45:e18. 10.1093/nar/gkw955 PMC5389512 28204566

[CIT0003] Katoh K, Standley DM. 2013. MAFFT multiple sequence alignment software version 7: improvements in performance and usability. Mol Biol Evol. 30(4):772–780. 23329690 10.1093/molbev/mst010 PMC3603318

[CIT0004] Li B, Chen H, Chen J. 2020. The complete chloroplast genome of plant *Rrhamnus heterophylla* (Rhamnaceae). Mitochondr DNA B. 5(2):1850–1851.

[CIT0005] Stamatakis A, Hoover P, Rougemont J. 2008. A rapid bootstrap algorithm for the RAxMLweb-servers. Syst Boil. 57(5):758–771. 10.1080/10635150802429642 18853362

[CIT0006] Tillich M, Lehwark P, Pellizzer T, Ulbricht-Jones ES, Fischer A, Bock R, Greiner S. 2017. GeSeq - versatile and accurate annotation of organelle genomes. Nucleic Acids Res. 45(W1):W6–W11. 28486635 10.1093/nar/gkx391 PMC5570176

[CIT0007] Zhu XF, Li Y, Lu ZQ. 2019. The complete chloroplast genome sequence of *Berchemia flavescens* (Rhamnaceae). Mitochondr DNA B. 4(1):1302–1303.

